# State Regulations to Support Children’s Cultural and Religious Food Preferences in Early Care and Education

**DOI:** 10.1007/s10995-019-02833-1

**Published:** 2019-12-12

**Authors:** Anna Ayers Looby, Natasha Frost, Sarah Gonzalez-Nahm, Elyse R. Grossman, Julie Ralston Aoki, Sara E. Benjamin-Neelon

**Affiliations:** 1 University of Minnesota Medical School, Duluth Campus, Duluth, MN 55812 USA; 2 Public Health Law Center, Mitchell Hamline School of Law, St Paul, MN 55105 USA; 3 Department of Health, Behavior and Society, Johns Hopkins Bloomberg School of Public Health, Baltimore, MD 21205 USA; 4 Lerner Center for Public Health Promotion, Johns Hopkins Bloomberg School of Public Health, Baltimore, MD 21205 USA

**Keywords:** Child care, Culture, Policy, Regulations

## Abstract

**Objective:**

In July 2018 the Academy of Nutrition and Dietetics released a benchmark encouraging early care and education (ECE) programs, including child care centers and family child care homes, to incorporate cultural and religious food preferences of children into meals. We examined the extent to which states were already doing so through their ECE licensing and administrative regulations prior to the release of the benchmark. This review may serve as a baseline to assess future updates, if more states incorporate the benchmark into their regulations.

**Methods:**

For this cross-sectional study, we reviewed ECE regulations for all 50 states and the District of Columbia (hereafter states) through June 2018. We assessed consistency with the benchmark for centers and homes. We conducted Spearman correlations to estimate associations between the year the regulations were updated and consistency with the benchmark.

**Results:**

Among centers, eight states fully met the benchmark, 11 partially met the benchmark, and 32 did not meet the benchmark. Similarly for homes, four states fully met the benchmark, 13 partially met the benchmark, and 34 did not meet the benchmark. Meeting the benchmark was not correlated with the year of last update for centers (*P *= 0.54) or homes (*P *= 0.31).

**Conclusions:**

Most states lacked regulations consistent with the benchmark. Health professionals can help encourage ECE programs to consider cultural and religious food preferences of children in meal planning. And, if feasible, states may consider additional regulations supporting cultural and religious preferences of children in future updates to regulations.

## Significance

*What is already known on this subject?* A culturally competent child care program can contribute to the health of all children in care. In July 2018, the Academy of Nutrition and Dietetics released a benchmark encouraging early care and education (ECE) programs to incorporate cultural and religious food preferences of children into meals.* What this study adds?* We examined the extent to which states were already doing so through their ECE licensing and administrative regulations prior to the release of the benchmark to serve as a baseline. We found that few states had regulations consistent with the benchmark to incorporate cultural and religious food preferences of children into meal planning.

## Introduction

It is important to consider cultural food preferences to improve dietary intake in young children in the United States (US) and globally (Chege et al. 2015). There is evidence that cultural food preferences are reflected in maternal feeding practices as early as infancy (Hardison-Moody et al. 2018). But, children’s development and related feeding practices are not occurring in isolation within the family home. Early care and education (ECE) settings are an important part of children’s development. Out-of-home child care is common and large numbers of young children spend time in ECE ((UNICEF), 2008). Over half of US children under five years are in regular child care (12 million or 61 percent) (Laughlin, 2013). Children may consume a large percentage of their daily nutritional requirements in care, which emphasizes the importance of the ECE setting (Neelon and Briley, 2011).

A culturally competent ECE program may contribute to the health and enrichment of all children. Child care providers play an important role in ensuring that children have access to healthy foods in ECE, and should provide foods that reflect children’s cultural and religious backgrounds (Neelon and Briley, 2011; Larson et al. 2011). Research shows that children in ECE are more likely to accept and consume familiar foods and those consistent with foods provided at home (Mazarello Paes et al. 2015). Cultural and environmental factors also play a role in parents’ involvement in their children’s ECE programs (Mena et al. 2015). Child care providers can engage in a larger dialogue around healthy food access and health equity. Access to culturally relevant, healthy foods is a part of children’s early foundation. Thus, it is important for child care providers to consider children’s culture, religion, and other preferences when planning food provided in care.

In July 2018, the Academy of Nutrition and Dietetics put forth a benchmark encouraging ECE programs to incorporate the cultural and religious food preferences of children into meal planning (Benjamin-Neelon, 2018). We examined the extent to which states included this requirement through their ECE licensing and administrative regulations prior to the release of the benchmark. This review may serve as a baseline to assess future updates as more states consider and incorporate the benchmark into their regulations.

## Methods

### Benchmark

In July 2018, the Academy of Nutrition and Dietetics released its new position paper—benchmarks for nutrition in ECE for children ages two to five years (Benjamin-Neelon, 2018). The 12 new benchmarks provide guidance for food and nutrition practitioners, parents, and providers; one benchmark encourages the provision of cultural foods and respect for culture in ECE. Previous Academy of Nutrition and Dietetics position papers for nutrition in ECE from 2005 (“Position of the American Dietetic Association: benchmarks for nutrition programs in child care settings,”^2005^) and 2011 (Neelon and Briley, 2011) have referenced cultural and religious food preferences of children, but neither included an explicit benchmark. Researchers have assessed the extent to which these benchmarks have been incorporated into ECE practice (Dev et al. 2017; Dev and McBride, 2013), but not policy or regulation. We evaluated the extent to which states required ECE programs to incorporate the cultural and religious food preferences of children into meal planning through state regulation.

### Review of Regulations

For this cross-sectional study, we reviewed state regulations for ECE facilities for all 50 US states and the District of Columbia contained on a federal government website through June 2018 – prior to the release of the new benchmark (U.S. Department of Health and Human Services 2018). We assessed consistency with the benchmark for both child care centers (hereafter centers) and family child care homes (hereafter homes). Centers are typically larger facilities in a dedicated building that serve a greater number of children, whereas homes are often located in a personal residence with the owner or renter as the primary provider of child care. Two independent reviewers examined state regulations using a combination of full text review and key word searches. Regulations were considered as partially or fully meeting the benchmark if they contained language related to culture, ethnicity, or religion regarding foods served at the ECE program; parental preferences or requests of meal pattern modification; or general understanding of or respect for cultural or religious differences in the context of meals and snacks. To be coded as fully meeting the benchmark, regulations had to explicitly require ECE programs to provide foods that reflected children’s culture, ethnicity, or religion, rather than accommodating specific parent requests. Regulations were deemed to partially meet the benchmark if they required menu modification in response to a parent request due to special dietary, religious, or cultural needs of specific children. Agreement between the reviewers was 74.8%.

### Analysis

We categorized states as fully meeting, partially meeting, or not meeting the benchmark encouraging child care programs to incorporate children’s cultural and religious preferences into meal planning for both centers and homes.

## Results

### Child Care Centers

Eight states had regulations that fully met the benchmark for child care centers, including Connecticut, Hawaii, Illinois, Kansas, Michigan, Mississippi, Nevada, and North Dakota (Table 1). Additionally, 11 states had regulations that partially met the benchmark and 32 did not meet the benchmark. Of the 19 states that fully or partially met the benchmark, the specific wording of regulations varied. For example, the regulation for Illinois child care centers fully met the benchmark in Ill. Admin. Code Tit. 89, Pt. 407.40, stating “[m]enu planning shall reflect consideration for cultural and ethnic patterns.” Similarly, the regulation governing North Dakota centers fully met the benchmark in N.D. Admin. Code 75-03-11.1-20 and states, “The cultural diversity of the children must be reflected in the program through incorporation of their language, food, celebration, and lifestyles, if appropriate.” Conversely, the regulation for South Carolina partially met the benchmark in S.C. Code Regs. 114-508 and states, “[d]ietary alternatives shall be available for a child who has special health needs or religious beliefs.” The regulation for Maryland also partially met the benchmark in Md. Code Regs. 13A.17.12.02 and states, “[i]f an operator agrees to accept a child who requires a modified diet for cultural or religious reasons, the operator shall obtain written, dated instructions for the diet signed by the child’s parent.”

**Table 1:** State regulations that fully met, partially met, or did not meet the benchmark to incorporate cultural and religious food preferences of children into meal planning through June 2018

State	Regulation fully met benchmark	Regulation partially met benchmark	Regulation did not meet benchmark
Centers	Connecticut; Hawaii; Illinois; Kansas; Michigan; Mississippi; Nevada; North Dakota	District of Columbia; Georgia; Maryland; New Hampshire; New Jersey; New York; North Carolina; Ohio; South Carolina; Vermont; West Virginia	Alabama; Alaska; Arizona; Arkansas; California; Colorado; Delaware; Florida; Idaho; Indiana; Iowa; Kentucky; Louisiana; Maine; Massachusetts; Minnesota; Missouri; Montana; Nebraska; New Mexico; Oklahoma; Oregon; Pennsylvania; Rhode Island; South Dakota; Tennessee; Texas; Utah; Virginia; Washington; Wisconsin; Wyoming
Homes	Hawaii; Mississippi; Nevada; Washington	Colorado; Connecticut; District of Columbia; Illinois; Maryland; New Hampshire; New York; North Carolina; North Dakota; Ohio; South Carolina; Vermont; West Virginia	Alabama; Alaska; Arizona; Arkansas; California; Delaware; Florida; Georgia; Idaho; Indiana; Iowa; Kansas; Kentucky; Louisiana; Maine; Massachusetts; Michigan; Minnesota; Missouri; Montana; Nebraska; New Jersey; New Mexico; Oklahoma; Oregon; Pennsylvania; Rhode Island; South Dakota; Tennessee; Texas; Utah; Virginia; Wisconsin; Wyoming

### Family Child Care Homes

Four states had regulations that fully met the benchmark for homes, including Hawaii, Mississippi, Nevada, and Washington. Thirteen other states had regulations that partially met the benchmark and 34 states had regulations that did not meet the benchmark (of note, Louisiana does not regulate family child care homes and therefore did not meet the benchmark). The regulation for homes in Nevada (Nev. Admin. Code § 432A.380) fully met the benchmark stating, “[c]ultural and ethnic foods which are appropriate for children must be considered in planning meals.” In Mississippi, the regulation (Miss. Code R. § 15-16-1:3.13.2) fully met the benchmark, stating, “[f]oods shall be provided in quantities and meal patterns that balance energy and nutrients with children’s … cultural and ethnic differences in food habits.”

On the other hand, the regulation governing Ohio homes (Ohio Admin. Code 5101:2-13-22) partially met the benchmark, stating, “[w]hen special diets are required for cultural or religious reasons, the … home shall obtain written … instructions from the child’s parent or guardian ….” In North Carolina, the regulation for homes (10A N.C. Admin. Code 09.1706) partially met the benchmark, stating, “[t]he food required by special diets for medical, religious or cultural reasons, may be provided … or may be brought … by the parents.”

## Discussion

Our review of the ECE regulations revealed that most states did not have regulations consistent with the benchmark to incorporate the cultural and religious food preferences of children into meal planning. There were, however, some states (Hawaii, Mississippi, and Nevada) that had regulations that fully met the benchmark for both centers and homes. Generally, we observed stronger regulations for centers compared to homes. This finding is consistent with previous reviews examining the presence and strength of state regulations for ECE related to infant feeding (Benjamin et al. 2009) and breastfeeding (Gonzalez-Nahm et al. 2017). Overall, centers tend to be regulated more often and more stringently than homes. Few states, however, had regulations that required centers or homes to fully consider the cultural and religious preferences of children in their meal planning. The results of this review, however, may serve as a baseline to assess future changes in state regulations and consistency with the 2018 benchmark, especially as ECE programs become greater targets for the promotion of culturally and religiously appropriate foods.

Though there has been growing national attention to strengthening the ECE systems to support healthy child development, more can be done to appropriately support culturally and religiously appropriate foods in these settings. Food practices are frequently steeped in cultural tradition; oftentimes meals are incorporated into ceremonies and are used as a marker to help define a social group or identity (Block et al. 2011). The many heterogenous American Indian/Native Alaskan (AI/NA) and immigrant populations provide powerful examples to consider in dialogues around access to cultural and religious foods. Reinforcing traditional foods and cultural practices is a promising approach to address the disproportionately high rates of diabetes experienced by many AI/NA groups (Satterfield et al. 2016). Some research suggests that the nutrition transition that AI/NA populations experienced over the course of history is partially responsible (Story et al. 1998; Welty, 1991). A prior study also found that the Westernization of native diets can affect markers of cardiovascular health (Bersamin et al. 2008). However, culturally-tailored interventions can help improve dietary habits and manage chronic disease in diverse populations (Juarez-Ramirez et al. 2019; Seguin et al. 2019; Story et al. 1999). Similarly, the epidemiological paradox exists, where newcomers to the US tend to arrive in better health and have better health outcomes than their US-born children or grandchildren. This “healthy immigrant effect” is complicated, but one component is thought to be the cultural change in diet and dietary intake (Gushulak, 2007). As a part of this comprehensive approach, it is important to consider the barriers and opportunities that govern ECE environments, including the foods served to children.

Many children in the US are growing up in heterogenous, multicultural communities with cultural and religious blending. Recognizing this, we argue that state-level policy like an ECE regulation is still useful grounds for discussion and support of culturally and religiously appropriate ECE. Policy-based interventions have the potential to improve healthy eating in ECE and may be more sustainable than other types of behavioral interventions (Larson et al. 2011; Lessard and Breck, 2015). Moreover, a recent study highlighted the need for culturally sensitive policies within ECE (Messiah et al. 2017). There is also evidence that states use the benchmarks in drafting their regulations. Mississippi, for example, explicitly based its nutrition regulations for both centers and homes on the benchmarks in Miss. Admin. Code 15-11-55. Other states may consider incorporating the cultural foods benchmarks in future revisions or updates to regulations.

This study has limitations. Cross-sectional reviews do not address actual practices within ECE programs. Despite this, policy-based approaches to promoting healthy eating in ECE are becoming more common in the US (Neelon and Briley, 2011; Larson et al. 2011; Van Stan et al. 2013). Previous cross-sectional studies have documented nutrition-related regulations targeting ECE and found wide variation among states (Benjamin et al. 2008; Kim et al. 2012). However, a few studies have evaluated regulations and assessed provider practices or child health outcomes. One prior study quantified the potential health impact of a multicomponent regulatory intervention for ECE and found that regulatory changes could lead to decreased screen time, increased physical activity, and lower sugar-sweetened beverage consumption in children in care (Wright et al. 2015). In another prior study, researchers evaluated compliance with nutrition regulations in New York City (Lessard et al. 2014). They found that greater than 80% of ECE centers complied with the regulations governing milk, juice, and sugar-sweetened beverages. Additionally, about 50% complied with the juice and water regulations. However, the researchers evaluated compliance with the regulation after it was enacted and did not collect baseline data prior to the implementation of the regulation. Another previous study prospectively assessed compliance with new healthy eating regulations in a single state and found some improvement in alignment with the nutrition standard after the policy took effect (Neelon et al. 2016). Researchers evaluated 13 nutrition standards governing ECE centers serving low-income children in South Carolina and used North Carolina, a state not making policy changes, as the comparison (Neelon et al. 2016). They found that the new standards modestly improved nutrition practices in South Carolina centers post-policy. Thus, although there is evidence that state regulations have the potential to impact nutrition practices, more information is needed to demonstrate the effectiveness of new or enhanced regulations in a variety of ECE settings and states.

Early care and education programs could also incorporate the cultural and religious preferences of children into meal planning without being prompted by regulation, and this review would not capture these practices. However, there is no specific funding mechanism for states or for ECE programs to encourage implementation of the standards. The standards are also not disseminated directly to states or programs. Despite this, ECE programs may still be engaging in meal planning that aligns with the standard. Another limitation is that this study did not include Tribal laws or local jurisdictional ECE licensing laws. Tribal licensing laws and local licensing laws were outside the scope of our analysis, although these laws also merit examination as many Tribes have laws regulating child care programs within their jurisdictions. Additionally, this review is current through June 2018 and states may have already updated their regulations. Researchers interested in monitoring and evaluating ECE regulations should consider conducting these reviews regularly to assess change over time.

## Conclusions for Practice

State licensing and administrative regulations for ECE programs have the potential to support the cultural and religious preferences of children in care. However, the support for culturally and religiously appropriate foods in ECE remains underdeveloped. If feasible, states may consider additional regulations supporting cultural and religious preferences of children in meal planning in future updates to regulations. When regulation is not yet possible, states with a Quality Rating and Improvement Systems (QRIS) could incorporate the standard. A QRIS is a voluntary but systematic approach to assess, enhance, and communicate the quality of ECE programs in a given state; ECE programs can opt to participate but must meet certain standards. Currently, about half of states have a QRIS (QRIS Resource Guide 2019). Finally, in the absence of regulations and a QRIS system, nutrition and health professionals can encourage ECE programs to consider the cultural and religious food preferences of children in meal and menu planning.
